# Construction and Comparison of Molecular Identification Methods for *Mannheimia haemolytica*

**DOI:** 10.3390/microorganisms14091998

**Published:** 2026-09-09

**Authors:** Zhen Yang, Yongqiang Miao, Hao Mou, Suhui Zhang, Liu Yang, Dengfeng Xu, Lizhi Fu, Kefei Shen, Ziqi Li, Long Zhao, Yuandi Yu

**Affiliations:** 1 National Technology Innovation Center for Pigs, Chongqing 402460, China; yangz@cqaa.cn (Z.Y.); miaoyq@cqaa.cn (Y.M.);; 2 Chongqing Academy of Animal Sciences, Chongqing 402460, China; 3 Rongchang Field Scientific Observation and Research Station for Animal Diseases, Ministry of Agriculture and Rural Affairs, Chongqing 402460, China; 4 College of Veterinary Medicine, Southwest University, Chongqing 402460, China

**Keywords:** *Mannheimia haemolytica*, PCR identification, *rpt2* primer, clinical detection

## Abstract

*Mannheimia haemolytica* is a primary pathogen causing bacterial pneumonia in cattle and sheep. Multiple PCR targets exist for its identification, but no standardized primer pairs have been widely adopted. This study evaluated the universality of six reported primers (including *rpt2*) using clinical *M. haemolytica* isolates. Genus specificity was tested against standard strains of common ruminant pathogenic/commensal bacteria and other *Mannheimia* species. Four primer sets (*gcp*, *artJ-lktC*, *lktD*, *sodA*) failed to distinguish *M. haemolytica* from other *Mannheimia* species. The *rpt2* primer showed suboptimal universality, with 3 of 22 tested isolates yielding negative results. The *rpoB* primer had favorable universality and specificity, but its 136 bp amplicon was unsuitable for constructing multiplex PCR with *M. haemolytica* serotyping primers. We identified design flaws in the original *rpt2* primer and developed a novel *rpt2-N* primer. Evaluation confirmed its strong universality, specificity, and sensitivity, as well as compatibility with serotyping primers for multiplex PCR assays. Field testing on three goat farms in the Chongqing region showed markedly higher *M. haemolytica* positivity rates after sharp temperature drops and transportation. Serotype A2 was confirmed as the dominant serotype in goats in Chongqing. This study systematically assessed multiple PCR identification primers, confirmed the feasibility of *rpoB*, and resolved the universality limitation of *rpt2*, providing technical support for rapid and accurate *M. haemolytica* detection.

## 1. Introduction

*Mannheimia haemolytica*, previously named *Pasteurella haemolytica*, belongs to the genus *Mannheimia* within the family *Pasteurellaceae* [1]. This genus covers multiple species, including *Mannheimia haemolytica*, *Mannheimia granulomatis*, *Mannheimia ruminalis*, *Mannheimia succiniciproducens*, *Mannheimia glucosida*, *Mannheimia varigena*, and *Mannheimia nigrescens* [2]. As the most clinically prevalent species in this genus, *M. haemolytica* primarily colonizes the upper respiratory tracts of ruminants such as cattle, goats and sheep. Host immunity declines under pathogen invasion or adverse environmental stress, enabling rapid proliferation of *M. haemolytica* in vivo [3,4]. This bacterium acts as a major etiological agent of the bovine respiratory disease complex and ovine pneumonia. Recently, *M. haemolytica*-associated abomasitis has been clinically described as a cause of sudden death in lambs [5]. Other *Mannheimia* species each have unique pathogenic traits. *M. glucosida* frequently triggers mastitis in sheep. By comparison, *M. succiniciproducens* and *M. ruminalis* normally live harmlessly within animal bodies and hardly lead to clinical illness [6]; however, Omaleki et al. isolated *M. ruminalis* from cases of acute mastitis in ewes [7].

Current identification methods for *M. haemolytica* mainly include conventional pathogen isolation and biochemical testing [8,9,10], matrix-assisted laser desorption/ionization time-of-flight mass spectrometry (MALDI-TOF MS) [11,12], and PCR-based molecular assays [13,14]. Conventional identification relies on observing strain growth profiles and hemolytic activity on selective agars such as sheep blood agar and MacConkey agar, along with Gram-staining morphology and biochemical profiling for final species confirmation. Biesheuvel et al. documented that biochemical assays and MALDI-TOF MS deliver definitive identification for most isolates [15]. Both techniques, however, require prior single-colony isolation and cannot test raw clinical specimens directly. PCR assays work well on purified bacterial cultures and tissue samples alike, enabling rapid screening for host infection with *M. haemolytica* [15]. However, no standardized molecular target has been established for the PCR-based identification of *M. haemolytica*. Documented target genes include *artJ-lktC*, *rpt2*, *lktD*, *rpoB*, *gcp*, and* sodA* [14,16,17,18,19,20].

Primers targeting the *rpt2* gene are the most widely used tools to distinguish *M. haemolytica* isolates and show high specificity in published trials. Upon reviewing electrophoretic data from previous publications, our team noticed this primer set could not identify certain field isolates [13]. Researchers have developed multiplex PCR assays relying on the *sodA* gene to identify *M. haemolytica* and differentiate this pathogen from other bovine respiratory bacteria [16]. Still, no research has validated the specificity of these assays against other *Mannheimia* species colonizing the upper respiratory tract of small ruminants. Multiple *Mannheimia* species carry leukotoxin-associated genes, such as *lktA* and *lktD* [17]. The *rpoB* molecular target was newly designed in 2024, and further evaluation is needed to confirm the reliability of its corresponding primers [14]. Similarly, the *artJ-lktC* and *gcp* primers have not been systematically evaluated as identification targets.

Hence, our study compared the specificity and universality of six previously reported PCR primer sets for *M. haemolytica* detection, with a particular focus on the *rpt2* primer. To address its poor universality, we redesigned the target region of the *rpt2* gene and further validated its specificity and sensitivity. We subsequently developed a multiplex PCR assay by pairing the optimized primer with validated serotyping primers. Subsequently, this optimized primer was applied to screen for *M. haemolytica* in samples from multiple goat farms and free-range flocks across Chongqing. The findings are expected to enable accurate diagnosis of *M. haemolytica* infection and reduce its adverse impacts on the cattle and sheep breeding industries.

## 2. Materials and Methods

### 2.1. Bacterial Isolates/Strains

In this study, the term “isolate” refers to *M. haemolytica*, other members of the genus *Mannheimia*, and *Bibersteinia trehalosi* recovered from field samples during our previous epidemiological survey. The term “strain” refers specifically to standard or reference strains used for evaluating assay specificity.

A total of 22 archived *M. haemolytica* isolates preserved in our laboratory were included in this study. These strains were isolated from healthy or respiratory-diseased animals in Chongqing, China, including 16 goat-derived isolates and six cattle-derived isolates. These isolates contained two serotype A1 isolates, 16 serotype A2 isolates, and two serotype A6 isolates (Table 1). All isolates were cultured on LB or LB agar (Difco, Franklin Lakes, NJ, USA), supplemented with 5% newborn bovine serum (Solarbio, Beijing, China), at 37 °C under a humidified atmosphere with 5% CO_2_.

To evaluate the intra-genus specificity of the established PCR assay, four *Mannheimia* species (*M. glucosida*, *M. granulomatis*, *M. ruminalis*, and *M. varigena*) were tested. In addition, a total of 18 bacterial strains, consisting of closely related species, and common pathogens colonizing the respiratory tracts of herbivores, like *Klebsiella pneumoniae* and *Moraxella ovis*, were included for specificity verification (Supplementary Table S1).

### 2.2. Sampling

#### 2.2.1. Preliminary Evaluation of Swab Types

Before formal sampling, we first evaluated the applicability of nasal and pharyngeal swabs for detecting *M. haemolytica*. In two pens of a goat farm in Dazu District, Chongqing, 20 and 15 goats were randomly selected for pharyngeal and nasal swab sampling, respectively. With veterinary assistance, animals were moderately restrained prior to sampling. The external nares were disinfected with 75% ethanol, and a sterile cotton swab (20–25 cm in length, CY-96000, Huachenyang Technology, Shenzhen, China) was inserted into the nasopharynx via the ventral nasal meatus, gently rotated three times, and withdrawn. Following nasal swab collection, the external mouth and perioral region were disinfected with 75% ethanol. The oral cavity was gently opened, and a sterile cotton swab (CY-98000, Huachenyang Technology, Shenzhen, China) was advanced along the dorsal tongue surface into the deep pharynx (approximately 8–12 cm) and rotated three times against the posterior pharyngeal wall and tonsillar area to maximize recovery of mucosal secretions. Swabs were then placed in sterile tubes containing 2 mL of DNA preservation solution (RK112, Tiangen, Beijing, China), sealed, and transported to the laboratory for analysis.

#### 2.2.2. Epidemiological Survey

**Figure 1:**
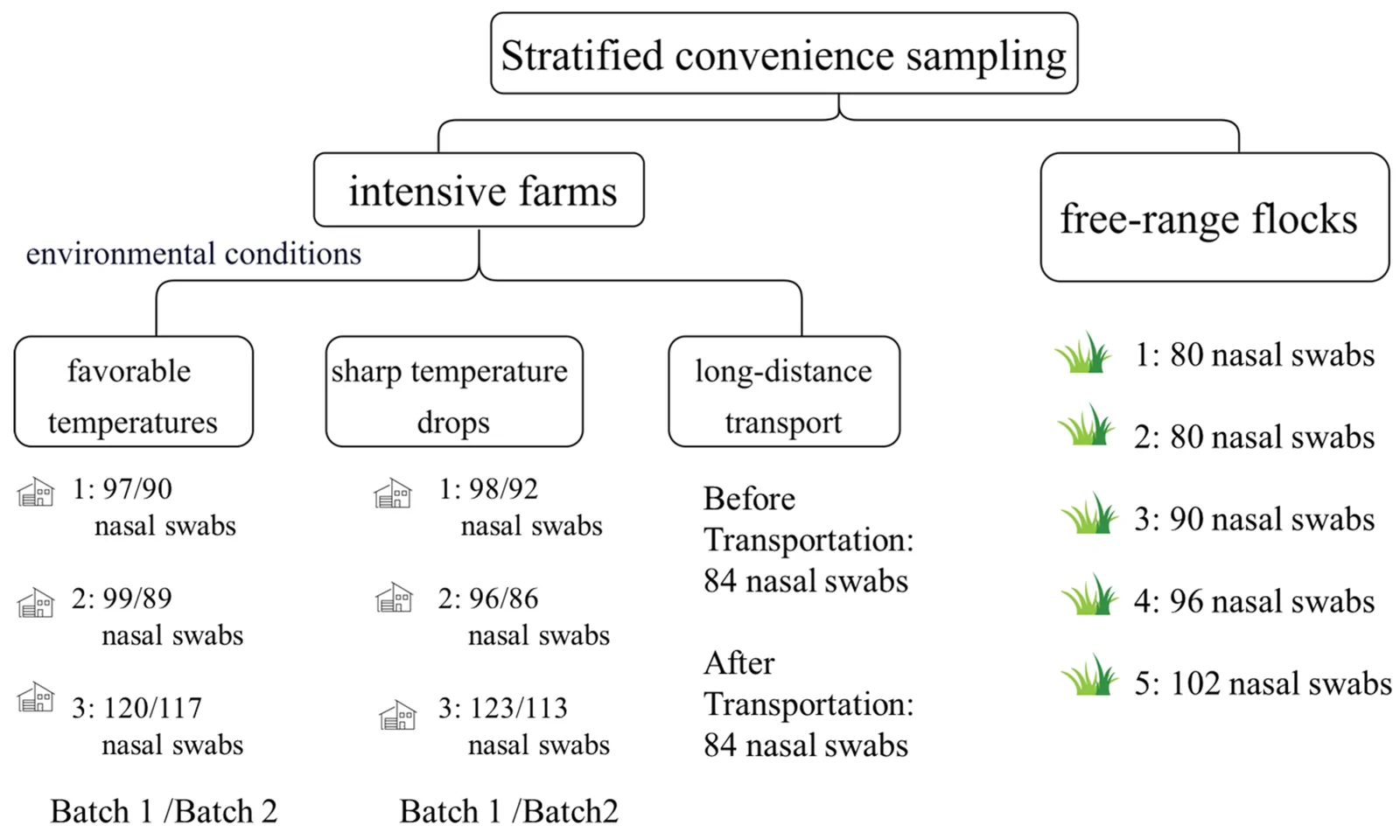
Sampling strategy and sample collection statistics. A stratified convenience sampling method was adopted in Chongqing, China, including 3 intensive goat farms and 5 free-range goat flocks. For each intensive farm, two independent sampling batches (B1, B2) were conducted under each environmental condition: favorable temperature (daily average 15–25 °C, diurnal difference ≤ 5 °C), sharp temperature drop (≥8 °C decrease within 24 h). For the long-distance transport group (>4 h), paired samples were collected before and after transport.

Subsequently, an epidemiological survey of *M. haemolytica* was conducted in Chongqing. Nasal swabs were collected using a site-level convenience sampling strategy from three intensive farms and five free-range flocks accessible to the research team. Goats with no apparent clinical abnormalities and no antibiotic administration within one week prior to sampling were enrolled. Within each site, sampling was stratified by environmental condition-favorable temperatures, sharp temperature drop, and long-distance transport to assess the impacts of environmental stressors on *M. haemolytica* colonization. As this was a field-based cross-sectional survey, no pre-specified sample size calculation was performed. For intensive farms, two independent sampling batches were conducted at each farm under each environmental stress condition. In total, 612 nasal swabs were collected in the favorable-temperature group and 608 in the sharp-temperature-drop group. For the long-distance transport group, 168 paired nasal swabs were obtained (84 before and 84 after transport). Nasal swabs were collected at 1 day before transport and at 3 days after transport. An additional 448 nasal swabs were collected from five free-range flocks. Overall, 1836 nasal swabs were included in this study. Sample sizes for each sampling event are detailed in Figure 1, and the criteria defining favorable temperatures and sharp temperature drops are provided in the figure legend.

### 2.3. DNA Isolation

In the present study, DNA extraction was performed from bacterial cultures, nasal swabs, and pharyngeal swabs using commercial genomic DNA isolation kits, according to the manufacturer’s instructions. Bacterial genomic DNA was extracted using the TIANamp Bacteria DNA Kit (DP302, Tiangen, Beijing, China). For goat nasal and pharyngeal swab samples, three additional kits, including the E.Z.N.A. Bacterial DNA Kit (D3350-00, Omega Bio-Tek, Norcross, GA, USA), Bacteria Genomic DNA Extraction Kit (CW0552S, CWBIO, Beijing, China), and Bacteria Genomic DNA Extraction Kit (YZ-D3350, Solarbio, Beijing, China), were also used, and the DNA extraction quality among different kits was subsequently compared. DNA quality was assessed by PCR amplification of target fragments using primers specific for the *rpt2* gene.

### 2.4. Primers and PCR Conditions

Six primers previously reported for PCR identification of *M. haemolytica* were subjected to species-specific testing in this study. The primer sequences and annealing temperatures are listed in Table 2. All PCR reactions were performed using a 25 μL mixture containing 2× rTaq enzyme (Cat. No. R001A, Takara Bio, Kusatsu, Shiga, Japan), 0.2 μM of each primer, and 100 ng of DNA template. Primer synthesis and Sanger sequencing were carried out by Sangon Biotech (Shanghai) Co., Ltd. (Shanghai, China).

### 2.5. Phylogenetic Analysis and Genotyping of the rpt2 Gene

The *rpt2* gene exhibits high inter-isolate sequence variability and has been reported to comprise three distinct genotypes [13]. Based on whole-genome sequencing data of *M. haemolytica* isolates generated in our previous study, the *rpt2* gene sequences were extracted from each isolate. The *rpt2* sequences of two representative isolates (Mh-22 and Mh-21) were used as queries for NCBI Web BLAST (https://blast.ncbi.nlm.nih.gov/Blast.cgi, accessed on 10 June 2024) against the GenBank database against the GenBank database. We retrieved *rpt2* sequences from *M. haemolytica* 39,433 (GenBank: CP017484.1) and the *rpt2*-divergent isolates NCTC 10,208 (GenBank: CP097335.1), 39,107 (GenBank: CP017490.1), and 0729 (GenBank: CP059327.1) based on the BLAST results. Phylogenetic trees were constructed using the neighbor-joining (NJ) method with the Kimura 2-parameter model in MEGA 11.0. The reliability of each branch was assessed by bootstrap analysis with 1000 replicates.

### 2.6. Development of PCR Assay

Concerning the insufficient universality of the existing *rpt2* primers, we screened the novel identification primer targets within the *rpt2* gene. First, the *rpt2* gene sequences from 22 *M. haemolytica* isolates were compared to delineate its conserved and variable regions. Then two candidate primer pairs (F1/R, F2/R) were designed in the conserved regions of the gene, and specificity testing on our *M. haemolytica* isolate panel identified the optimal pair, designated *rpt2-N*. All primers used in Section 2.4 are listed in Table 2. We optimized reaction conditions for *rpt2-N*, including annealing temperature, primer volume, reaction mixture, and Taq polymerase type. First, we observed the amplification efficiency at 50, 52, 54, 56, 58, 60, and 62 °C under the same reaction mixture to determine the optimal annealing temperature. Then, based on this temperature, we compared the amplification efficiencies of 10 μL, 15 μL, and 25 μL reaction mixtures, as well as 1 μL and 1.5 μL primer concentrations. We also tested amplification efficiency with two commercial Taq polymerases: Takara rTaq (Cat. No. R001A, Takara Bio, Kusatsu, Japan) and Vazyme Taq (Cat. No. P222-03, Vazyme Biotech, Nanjing, China).

### 2.7. Clinical Sample Detection

As described in Section 2.2, using *rpt2-N* primers, we detected the *M. haemolytica*-positivity rates for three farms and five free-range flocks in the Chongqing region. Based on the results from Section 2.5, PCR was performed in a 25 μL reaction mixture containing 1.5 μL of each primer and 50–100 ng template DNA. Amplification was performed with an initial denaturation step at 94 °C for 5 min, followed by 32 cycles consisting of 94 °C for 30 s, 52 °C for 30 s, and 72 °C for 1 min, and a final extension at 72 °C for 5 min.

### 2.8. Construction of Multiplex PCR

The PCR method established by Klima et al. was used to analyze the serotypes of *M. haemolytica* isolates [21]. Serotyping refers to the classification of *M. haemolytica* based on antigenic differences in surface capsular polysaccharides, and serotypes A1, A2, and A6 are the most prevalent among the known serotypes of *M. haemolytica*. Primers were designed, respectively, for three capsular antigen synthesis genes, namely *hyp*, *core* and *tupA*, and their sequences are listed in Table 3. To streamline the two-step detection procedure (bacterial species identification followed by serotyping), we combined *rpt2-N* primers with serotyping primers to establish a multiplex PCR assay. Each 25 μL multiplex PCR mixture contained 2× rTaq DNA polymerase (Cat. No. R001A, Takara Bio, Kusatsu, Shiga, Japan), serotyping primers at 0.1 μmol/L each, 0.2 μmol/L *rpt2-N* primers, and 100 ng of template DNA. The PCR protocol was set as follows: initial denaturation at 94 °C for 30 s; 32 cycles of denaturation at 94 °C for 30 s; annealing at 52 °C for 30 s; extension at 72 °C for 1 min; and a final extension at 72 °C for 5 min after cycling.

### 2.9. Statistical Analysis

Phylogenetic trees were made by MEGA v11 and pruned using the online server iTOLv4 (https://itol.embl.de/, accessed on 12 June 2024). The amino acid sequence alignment was performed with the ClustalX version 2.1 program and the ESPript 3.0 web tool (http://espript.ibcp.fr/ESPript/ESPript/, accessed on 12 June 2024). Graphs of *M. haemolytica* antigen positivity rates at each sampling site were generated using GraphPad Prism 8. To evaluate the effects of different rearing patterns on *M. haemolytica* positivity rates, data from six sampling rounds collected under ambient temperature conditions at three farms were compared with data from five sampling rounds of free-range flocks. To assess the impact of cold stress, the same dataset from the three farms was compared with data collected during sudden temperature drops. Differences between two groups were analyzed using Student’s *t* tests. Significant differences between groups are indicated using asterisks: * *p* < 0.05, ** *p* < 0.01, *** *p* < 0.001, and **** *p* < 0.0001; ns indicates not significant.

## 3. Results

### 3.1. Identification of M. haemolytica Field Isolates by PCR

**Figure 2:**
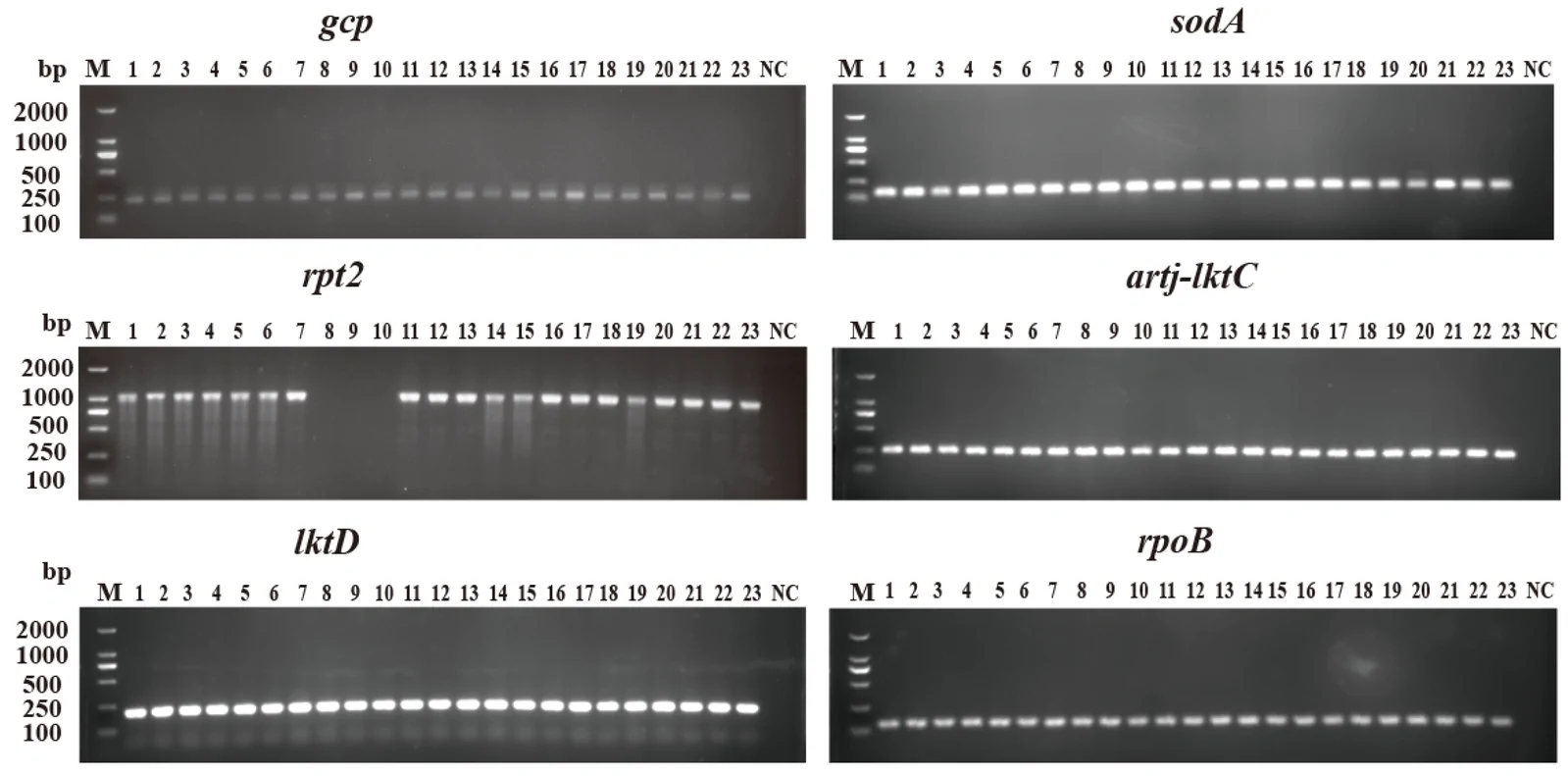
Agarose gel electrophoresis showing PCR products of each primer pair. Lanes: M = 2000 bp DNA marker; 1: positive control, genome of Mh39433; 2–23: genomes of *M. haemolytica* isolates; NC: negative control.

We used the isolates confirmed as *M. haemolytica* by sequencing to verify the accuracy of the six previously reported molecular identification primers for this bacterium individually. As shown in Figure 2, primers targeting the *gcp*, *rpoB*, *sodA*, *artJ-lktC* and *lktD* genes amplified the corresponding target bands in all 22 *M. haemolytica* isolates, while the primer for the *rpt2* gene yielded target fragments in 19 isolates, and slight variations were observed in amplicon lengths among different isolates.

### 3.2. Confirmation of Primer Specificity Against Closely Related Bacterial Species

Genomic DNA from four other *Mannheimia* species (excluding *M. haemolytica*) and two *Bibersteinia trehalosi* isolates served as templates to test the genus and species specificity of six *M. haemolytica* primer pairs. As shown in Figure S1, primers targeting *gcp*, *artJ*-*lktC*, *lktD*, and *sodA* generated non-specific amplicons matching the size and band brightness of the *M. haemolytica*-positive control. By contrast, the *rpt2* and *rpoB* primer sets produced no amplification bands under optimized PCR conditions. These data indicate that *rpt2* and *rpoB* primers possess strong specificity toward closely related bacterial species.

### 3.3. Confirmation of Amplification Efficiency and Specificity Against Non-Target Bacteria of M. haemolytica Primers

We focused on the primers targeting *rpoB* and *rpt2*, and further compared their amplification efficiency and species specificity for *M. haemolytica* using laboratory-preserved bacterial strains of bovine and ovine origin as templates. When tested against the 10 selected bacterial strains, the *rpt2* and *rpoB* primer pairs displayed robust species specificity with no non-target amplicons (Figure S2A). When genomic DNA from *M. haemolytica* strains served as the template, both primer sets had a limit of detection of 1 × 10^−3^ ng/μL (Figure S2B).

### 3.4. Molecular Characterization of the rpt2 Gene in M. haemolytica

**Figure 3:**
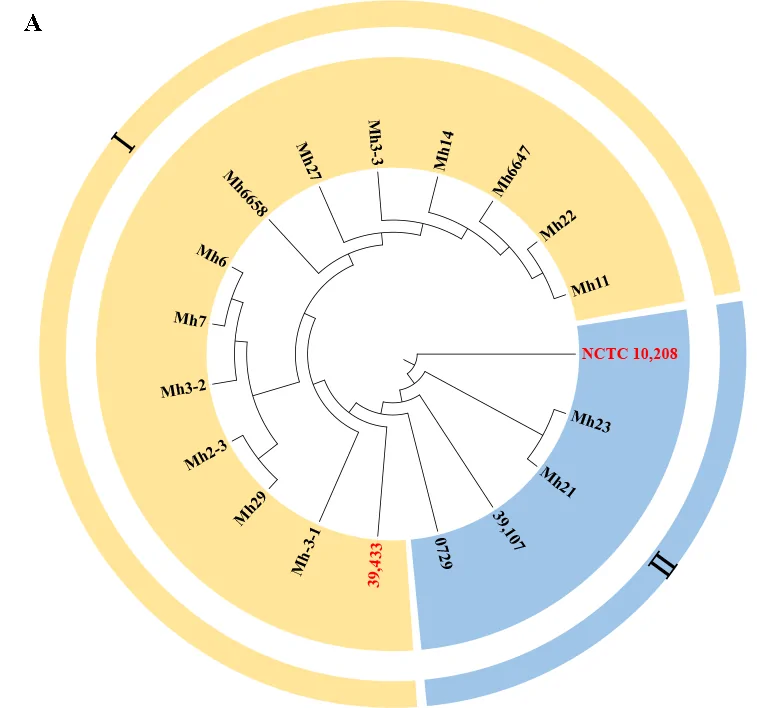

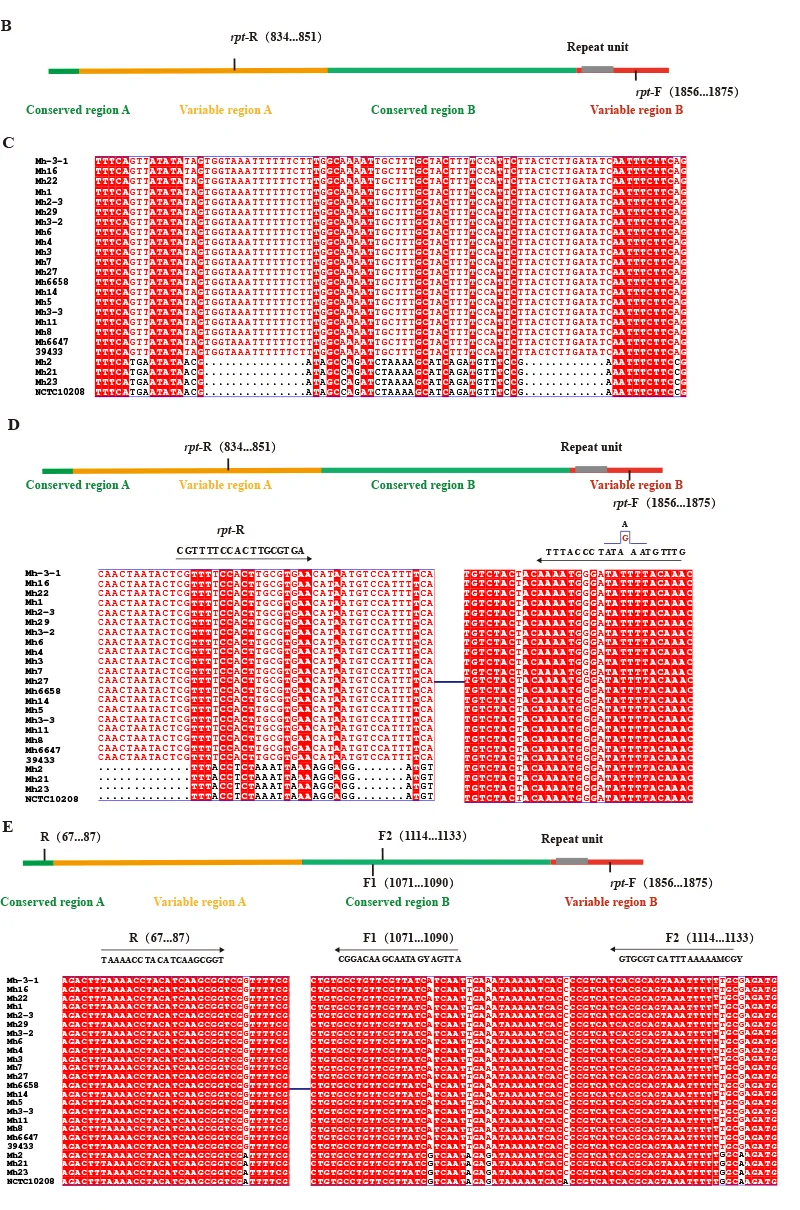
Characterization of the *rpt2* gene in *M. haemolytica*. (**A**) Phylogenetic tree based on the *rpt2* gene sequences of *M. haemolytica* strains. Blue indicates *rpt2* genotype I , and yellow indicates* rpt2 *genotype II. (**B**) Structural distribution of conserved and variable regions within the *rpt2* gene. *rpt*-F and *rpt*-R are the forward and reverse primers for *rpt2* amplification, mapping to 1856–1875 bp and 834–851 bp of the *rpt2* gene, respectively. The gray rectangle denotes GCACA tandem repeats within the *rpt2* gene. Dots in the sequences indicate base deletions at the corresponding positions of the *rpt2* gene in the tested isolate. (**C**) Partial sequence alignment of conserved region A of the *rpt2* gene from 18 *M. haemolytica* isolates. (**D**) Sequence comparison between the conventional *rpt2* amplification primers and the target sequences of different isolates. (**E**) Sequence matching validation of the two newly designed primer pairs (F1/R, F2/R) with *rpt2* gene sequences of the tested strains.

According to the above results, we decided to select *rpt2* as the target gene and redesigned the primers to achieve complete detection of *M. haemolytica*. We analyzed sequences of the *rpt2* gene. Phylogenetic analysis (Figure 3A) classified *M. haemolytica rpt2* into two genotypes. Most of the 14 isolates belonged to genotype I, represented by strain 39,433 . Only isolates Mh-21 and Mh-23 were genotype II, represented by strain NCTC 10,208, indicating significant genotypic divergence between the two genotypes. Sequence alignment identified two conserved regions and two variable regions within this gene (Figure 3B). Genotypic differentiation produced sequence variations in variable region A (Figure 3C). Polymorphisms in variable region B came from length changes in GCACA tandem repeats across strains. Conventional *rpt2* primers bind to variable regions and only match genotype I templates. Their reverse primers cannot anneal to genotype II templates, limiting assay universality (Figure 3D). Our new *rpt2* primers target conserved regions A and B. The reverse primer binds to conserved region A, and two forward primers bind to variable region B. Degenerate bases were added to forward primers to tolerate inter-strain base mismatches and improve assay performance (Figure 3E).

### 3.5. Performance of the PCR Assay

**Figure 4:**
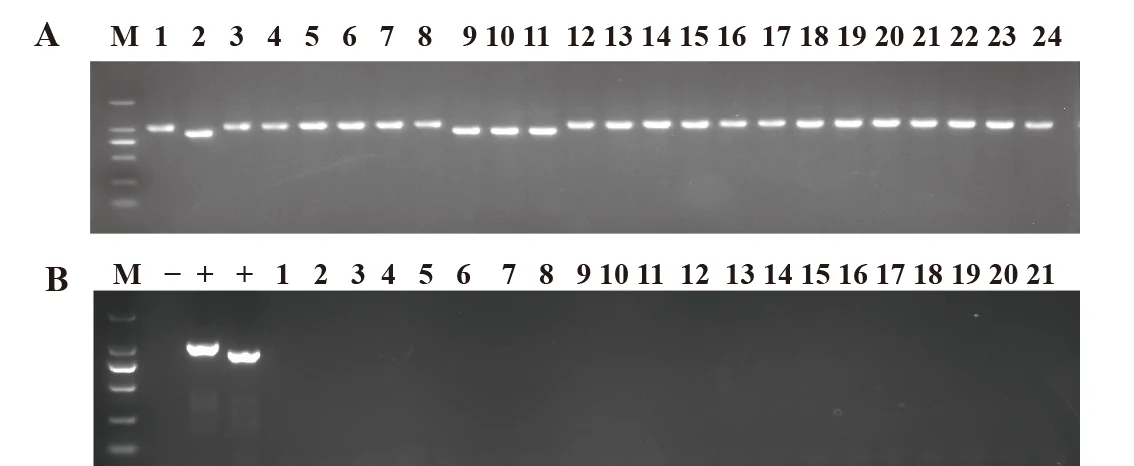
Specificity and universality of the *rpt2-N* primer. (**A**) Universality of the F2/R primer pair against 22 *M. haemolytica* isolates. Lanes: M = 2000 bp DNA marker; 1–2: positive control, genome of Mh 39,433 and genome of NCTC 10,208; 3–24: genomes of *M. haemolytica* isolates. (**B**) Further specificity validation of the F2/R primer pair. Lanes 1–21 correspond to genomic DNA from 21 preserved ruminant bacterial strains and isolates (Table S1).

We evaluated the species specificity of two newly designed primer pairs, F1/R and F2/R. Four non-specific bands were amplified using the F1/R primer pair, while no non-specific products were observed for F2/R (Figure S3A). The F2/R primers successfully identified all tested *M. haemolytica* isolates (Figure 4A). They amplified two distinct *rpt2* fragment sizes. Specifically, 19 isolates yielded a 1067 bp product of type I *rpt2*, and three isolates produced a 911 bp product of type II *rpt2*. This specific primer pair was designated *rpt2-N* to distinguish it from traditional *rpt2* primers. The optimized 25 μL PCR reaction mixture for *rpt2-N* contained 0.6 μM of each forward and reverse primer with an annealing temperature of 50 °C. This optimized PCR assay was specific for *M. haemolytica*, with no amplification from other *Mannheimia* species (Figure 4B). No positive amplicons were detected from other common bacterial strains isolated from cattle and ovines (Figure 4B). The limit of detection of the *rpt2-N*-based PCR was determined at the 40 copies/μL level (Figure S3B).

### 3.6. Multiplex PCR

A multiplex PCR assay was developed in this study using conserved *rpt-N* gene primers and serotype-specific primers to simultaneously identify *M. haemolytica isolate* and determine its serotypes. Electrophoresis data (Figure S4) exhibited specific amplicons corresponding to *rpt2-N* and serotype A2 marker Core2 genes in all 18 recovered *M. haemolytica* isolates. These findings validate the reliability of this multiplex PCR assay.

### 3.7. Clinical Detection

**Figure 5:**
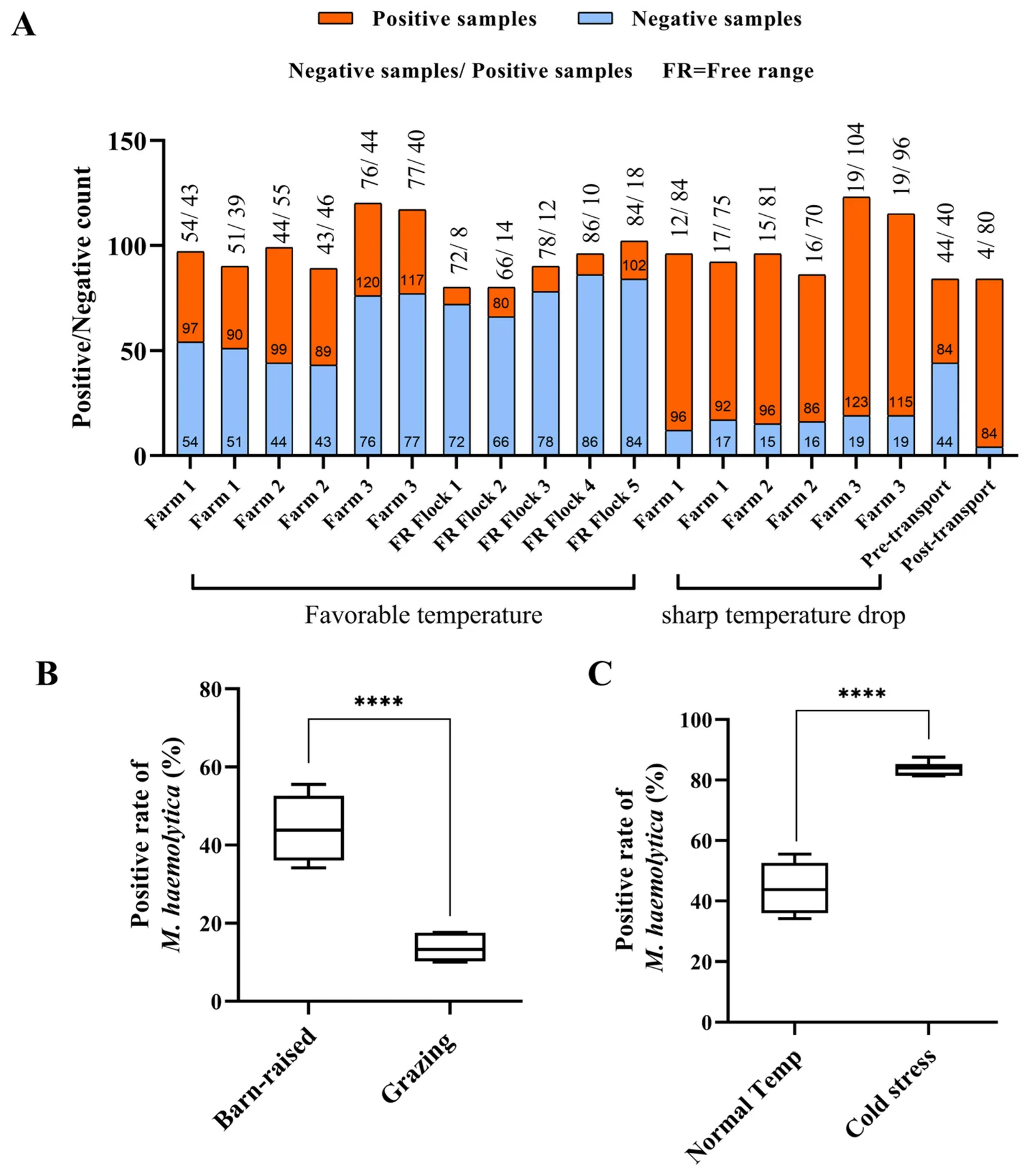
Epidemiological analysis of *M. haemolytica* in Chongqing goats using *rpt-N* primers. (**A**) Stacked bar chart showing the distribution of positive and negative *M. haemolytica* samples across different rearing environments, temperature conditions and transport stress conditions. (**B**) Stacked bars for positive/negative *M. haemolytica* samples by rearing mode. (**C**) Boxplots of *M. haemolytica* positivity rates for normal temperature and cold stress. , **** *p* < 0.0001; ns indicates not significant.

Clinical detection trials were performed to assess the field applicability of the *rpt*-N primers. Following comparison of sampling sites, DNA extraction kits, and amplification enzymes, we determined that bacterial genomic DNA extraction from nasal swabs using a commercial kit (Tiangen, Beijing, China), coupled with PCR amplification using rTaq DNA polymerase (Takara, Dalian, China), achieved satisfactory detection rates for *M. haemolytica* (Figure S5). All collected samples were subsequently screened for *M. haemolytica*. As illustrated in Figure 5A–C, goats raised on intensive farms exhibited a median *M. haemolytica* positivity rate of approximately 40%, whereas free-range goats showed a significantly lower positive rate of approximately 10% (*p* < 0.0001). Sharp temperature drop stress markedly elevated the *M. haemolytica* positivity rate to above 80% across all intensive farms (*p* < 0.0001). Furthermore, transport stress proved to be a significant risk factor, with the positive rate surging to 95.2% (80/84) in sheep following long-distance transportation (Figure 5A).

## 4. Discussion

Respiratory diseases have severely constrained the development of the global cattle, goat, and sheep breeding industries, with one of the key pathogens involved in this disease complex being *M. haemolytica* [22]. Biesheuvel reported that, in the Netherlands, fatal *M. haemolytica*-related deaths in dairy cows and veal calves rose by 1.5-fold and 1.4-fold, respectively, per 3-year period between 2004 and 2018 [11]. *M. haemolytica* isolation rates from ovine pneumonia lung tissues reached 21.8% (southwest Spain), 29.6% (north Ethiopia), and 34.2% (central Ethiopia) [13,23,24]. For diseased goats in northeast China and Sichuan, *M. haemolytica* pathogen detection rates reached 31.34% and 29.8%, respectively [25,26]. Given the severe pathogenicity of *M. haemolytica*, multiple PCR-based detection approaches for this pathogen have been established in previous studies. Nevertheless, a unified and standardized detection protocol is lacking currently.

Through a systematic evaluation of the universality and specificity of six existing identification primer pairs, we found that only the *rpt2* and *rpoB* primers could effectively distinguish *M. haemolytica* from other members of the genus *Mannheimia* and *B. trehalosi*. Notably, *B. trehalosi* was originally classified as biotype T of *M. haemolytica* and was later reclassified as an independent species based on molecular evidence; it is phylogenetically closely related to *M. haemolytica*. Dassanayake et al. used the *gcp* primers to differentiate these two bacterial species [20]. However, in the present study, the *gcp* primers failed to effectively distinguish between them when tested with two previously isolated *B. trehalosi* isolates. Given that only two isolates were available, we will obtain additional *B. trehalosi* isolates in future studies to further assess this finding.

*M. haemolytica* comprises 12 known serotypes, among which serotypes A1, A2, A6, and A7 predominate in field epidemiological surveys. Klima et al. established a multiplex PCR method to differentiate *M. haemolytica* serotypes 1, 2, and 6, generating amplicons of 306 bp, 160 bp, and 78 bp, respectively [21]. To achieve simultaneous detection of species identification and serotyping, we required a novel primer pair with an amplicon larger than 500 bp for the construction of the multiplex PCR system, ensuring effective differentiation from the serotyping primer amplicons during electrophoresis. The *rpoB* amplicon was only 136 bp, which was suitable for use in quantitative PCR for *M. haemolytica*, but inappropriate for developing a multiplex PCR system in combination with serotyping primers. Therefore, we focused on the *rpt2* gene itself. Analysis of the *rpt2* gene from 22 available *M. haemolytica* isolates revealed two distinct genotypes: 19 isolates belonged to genotype I and three to genotype II. The conventional *rpt2* reverse primer failed to match the genotype II template (Figure 3B–D), which explained the previous failure of molecular identification in these three isolates (Figure 2). BLAST analysis revealed that the vast majority of *M. haemolytica rpt2* genes were genotype I, with only a few being genotype II. This also explains why, in multiple published studies using *rpt2* for *M. haemolytica* identification, typically only two to four suspected isolates could not be molecularly identified. We designed novel identification primers targeting conserved region A and conserved region B of the *rpt2* gene. Notably, sequence alignment of genotype I and II *rpt2* alleles revealed two single-nucleotide mismatches within the forward primer binding region, located at the first and fourth positions from the 5′ end of the primer, respectively. We introduced degenerate bases Y (A/G) and M (T/G) at these two variable sites, thereby achieving efficient co-amplification of both genotypes in a single reaction system and ensuring primer universality (Figure 4). Due to a 156 bp deletion in the *rpt2* sequence of genotype II within the amplified region, identification using the *rpt2-N* primers generated two distinct band sizes: 1067 bp (genotype I) and 911 bp (genotype II) (Figure 4).

Aside from *rpt2*, the *rpoB* primers have been more comprehensively evaluated in the existing literature [14]. In terms of universality assessment, in addition to testing the common serotypes A1, A2, and A6, previous studies also tested one isolate of serotype A7, one of serotype A14, and six non-typeable isolates; these isolates were derived from goats, sheep, and cattle. In contrast, the universality assessment of *rpt2-N* in the present study focused primarily on serotypes A1, A2, and A6, as well as goat- and cattle-derived *M. haemolytica isolates*. Therefore, in future studies, we will further expand the collection of clinical samples from diverse sources to obtain more *M. haemolytica* isolates from different hosts and serotypes, thereby broadening the universality assessment of the *rpt2-N* primers. The site-level convenience sampling strategy employed in this study (samples collected only from three intensive farms and five free-range flocks accessible to the research team) has the following limitations. First, the number of selected farms and free-range flocks was limited. In addition, sites were selected purely based on accessibility to the research team, rather than random sampling. The enrolled intensive farms and free-range flocks may not represent the overall goat population in the Chongqing region across diverse production systems, biosecurity standards and management regimes, introducing obvious selection bias associated with geography and farming type. Finally, accessible sites may harbor unique disease backgrounds or management features, including prior infection history, customized vaccination schedules and medication routines. These limitations underscore the need for future studies to adopt randomized sampling across a wider range of farms and production systems to improve the representativeness of sampling.

Although *M. haemolytica* is an opportunistic pathogen, we contend that molecular detection remains necessary. Under physiological conditions, *M. haemolytica*, similar to other opportunistic pathogens, colonizes the upper respiratory tracts of goats at low bacterial loads; in the presence of specific stress factors, the bacterium undergoes significant proliferation and causes clinical disease in the host. Conventional PCR detection methods have limited sensitivity, whereas PCR-based positive detection rates can serve as practical indicators for assessing the health status of goat flocks. The clinical observations in the present study preliminarily support this notion: following sharp temperature drops or long-distance transport, the positive rate of *M. haemolytica* increased significantly; the positive rate in free-range flocks was considerably lower than that in intensive barn-raised flocks (Figure 5). Furthermore, the multiplex PCR detection method established in this study identified serotype A2 as the predominant epidemic strain of *M. haemolytica* in goats in Chongqing, providing foundational data for vaccine development against this pathogen.

Overall, we established a new *M. haemolytica* PCR detection method based on the *rpt2* gene, addressing the previous issue of primer specificity and confirming the feasibility of the *rpoB* primer.

## Figures and Tables

**Table 1:** List of *M. haemolytica* field isolates, and their host species, used in the current study.

Isolate	Host	Tissue	Year	Serovar
Mh-1	goat	Lung	2024	A2
Mh-2	cattle	Lung	2024	A2
Mh-3	goat	Nasal swab	2024	A2
Mh-4	goat	Lung	2024	A2
Mh-5	cattle	Nasal swab	2024	A2
Mh-6	goat	Lung	2024	A2
Mh-7	goat	Lung	2024	A2
Mh-8	goat	Lung	2024	A2
Mh-11	goat	Lung	2024	A2
Mh-14	cattle	Lung	2024	A1
Mh-15	cattle	Lung	2024	A1
Mh-16	cattle	Lung	2024	A6
Mh-21	goat	Nasal swab	2025	A2
Mh-22	cattle	Nasal swab	2025	A2
Mh-23	goat	Nasal swab	2025	A2
Mh-27	goat	Lung	2025	A6
Mh-29	goat	Nasal swab	2025	A2
Mh-2-3	goat	Nasal swab	2025	A2
Mh-3-1	goat	Lung	2025	A2
Mh-3-2	goat	Nasal swab	2025	A2
Mh-3-3	goat	Lung	2025	A2
Mh-6647	goat	Lung	2025	A2
Mh-6658	goat	Lung	2025	A2

**Table 2:** The primer pairs used in PCR for *M. haemolytica*.

Target Gene	Sequence (5′→3′)	Length of PCR Products (bp)	Annealing Temperature (°C)	Reference
*rpoB*	AACACATAAACGCCGTATCTCG	136	58	[14]
	GATATTCGGGCCTTCAGGA			
*sodA*	TTAGTATTAGAAGAGGGTAAATTAG	144	54	[16]
	AAATCGGATAGCCTGAAA			
*artJ-lktC*	TATAAGGATTACCACTTTAACGCA	251	60	[17]
	ATAATCAGAAGAGAAAGGAGTGT			
*rpt2*	GTTTGTAATATATCCCATTT	1022	50	[18]
	CGTTTTCCACTTGCGTGA			
*lktD*	GCAGGAGGTGATTATTAAAGTGG	206	56	[19]
	CAGCAGTTATTGTCATACCTGAAC			
*gcp*	AGAGGCCAATCTGCAAACCTCG	267	55	[20]
	GTTCGTATTGCCCAACGCCG			
F1/R	ATTGAYGATAACGAACAGGC	1024/868	56	This study
	TAAAACCTACATCAAGCGGTC			
F2/R	YGCMAAAAATTTACTGCGTG	1067/911	52	
	TAAAACCTACATCAAGCGGTC			

**Table 3:** Serotype identification primers for *M. haemolytica.*

Target Gene	Primers	Sequence (5′→3′)	Length of PCR Products (bp)	Annealing Temperature (°C)
*hyp*	Forward	CATTTCCTTAGGTTCAGC	306	55
	Reverse	CAAGTCATCGTAATGCCT		
*core2*	Forward	GGCATATCCTAAAGCCGT	160	55
	Reverse	AGAATCCACTATTGGGCACC		
*tupA*	Forward	TGAGAATTTCGACAGCACT	78	55
	Reverse	ACCTTGGCATATCGTACC		

## Data Availability

The original contributions presented in this study are included in the article/Supplementary Material. Further inquiries can be directed to the corresponding author.

## Supplementary Materials

The following supporting information can be downloaded at: https://www.mdpi.com/article/10.3390/microorganisms14091998/s1. Table S1. List of other non-M. haemolytica strains and isolates used in this study. Figure S1. Agarose gel electrophoresis showing PCR products of each primer pair. Figure S2. Amplification efficiency and specificity comparison of *rpoB* and *rpt2* primer sets. Figure S3. Universality and sensitivity of the rpt2-N primer. Figure S4. Gel electrophoresis results of the multiplex PCR system. Lanes 1 and 2: genome of Mh 39433 and genome of NCTC 10208 identified with *rpt2-N* primers. Lane 3: Mh 39433 detected *via Core* primers. Lane 4: Mh 39433 detected *via rpt2-N* primers and serotype primers. Lanes 5–22: Eighteen *M. haemolytica* isolates tested by multiplex PCR. Lane 23: Negative control. Figure S5. Epidemiological analysis of Mh in Chongqing sheep via *rpt2-N* primers.
