# Supplementary material for: Construction and Comparison of Molecular Identification Methods for Mannheimia haemolytica
Source: Microorganisms. 2026 Sep 9;14(9):1998. doi: 10.3390/microorganisms14091998 (PMC13609878; doi:10.3390/microorganisms14091998)
Supplement: Supplementary file 1 [file microorganisms-14-01998-s001.zip › microorganisms-4494289-supplementary.pdf]

Table S1. List of other non-*M. haemolytica* strains and isolates used in this study.

No.	Bacterial Species	Strain Number	GenBank Accession No.
1	*Staphylococcus aureus*	ATCC 25913	NR_111447.1
2	*Escherichia coli*	ATCC 25922	CP009072.1
3	*Acinetobacter baumannii*	CMCC 25154	NR_025333.1 / NZ_CP041700.1
4	*Klebsiella pneumoniae*	CMCC 46117	CP035202.1
5	*Corynebacterium pseudotuberculosis*	ATCC 19410	D38579.1 / NZ_CP021251.1
6	*Clostridium perfringens*	ATCC 13124	CP000246.1
7	*Pseudomonas aeruginosa*	ATCC 27853	NC_017549.1
8	*Mycoplasma mycoides subsp. capri*	F38, C87001	AF072715.1
9	*Pasteurella multocida* Serotype B	P52	EU016232.1 / PPVE00000000.1
10	*Trueperella pyogenes*	2012CQ-ZSH	CP012649.1
11	*Moraxella ovis*	CCTCC M2014036	JN084136.1
12	*Proteus mirabilis*	ATCC 43071	NC_010554.1
13	*Pasteurella multocida* Serotype A	HL	CP001409.1
14	*Streptococcus equi subsp. zooepidemicus* (SEZ)	ATCC 35246	NZ_CM001150.1
15	*Streptococcus equi subsp. zooepidemicus* (SEZ)	ST171	CP002904.1
16	*Streptococcus equi subsp. zooepidemicus* (SS2, Serotype 2 swine streptococcus)	HA9801	CP000872.1
17	*M. glucosida*	SZ-1	Clinically isolated from a goat farm, confirmed by 16S rRNA gene sequencing, and preserved in our lab
18	*M. glucosida*	SZ-2	
19	*M. granulomatis*	SZ-3	
20	*M. ruminalis*	SZ-4	
21	*M. varigena*	SZ-5	

22	*M. varigena*	SZ-6
23	*Bibersteinia trehalosi*	SZ-7
24	*Bibersteinia trehalosi*	SZ-8

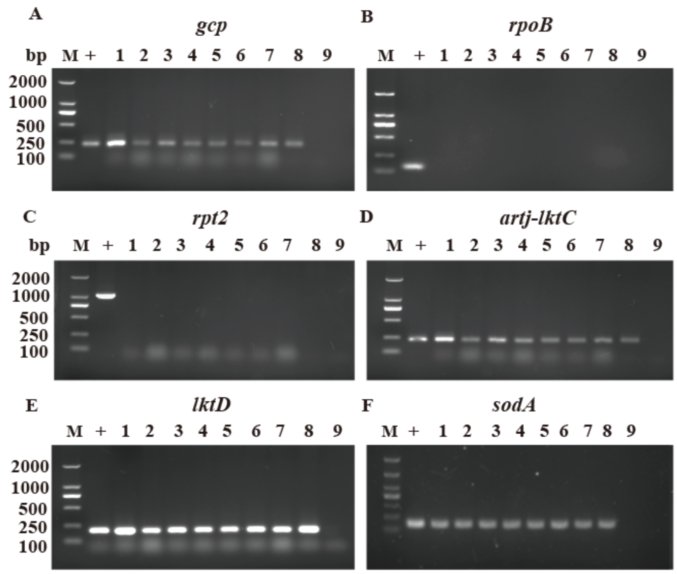

Figure S1. Agarose gel electrophoresis showing PCR products of each primer pair. (A) Primers targeting *gcp*; (B) Primers targeting *rpoB*; (C) Primers targeting *rpt2*; (D) Primers targeting *artJ-lktC*; (E) Primers targeting *lktD*; (F) Primers targeting *sodA*; Lanes: M=2000 bp DNA marker; +: M. haemolytica isolate Mh-3-1's genome; 1-2: *M. glucosida*'s genome; 3: *M. granulomatis*'s genome; 4: *M. ruminalis*'s genome; 5-6: *M. varigena*'s genome; 7-8: *B. trehalosi* 's genome; 9: Negative control.

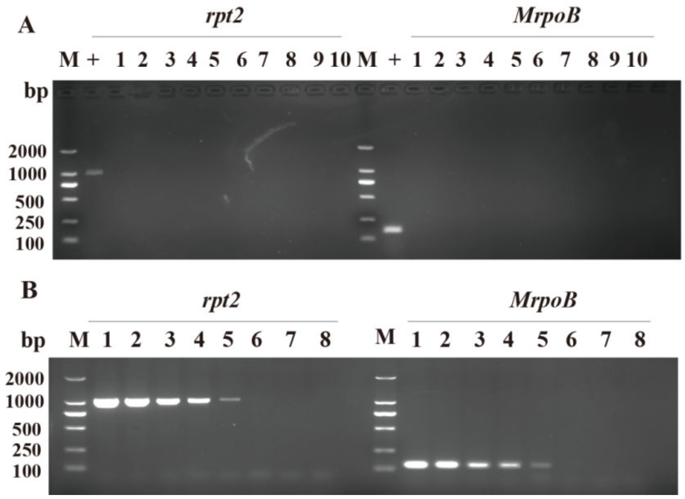

Figure S2. Amplification efficiency and specificity comparison of *rpoB* and *rpt2* primer sets. (A) Specificity verification of the two primer pairs. Lanes 1–10 correspond to genomic DNA from 10 ruminant strains stored in our lab (Table S1). (B) Agarose gel results of PCR using serially diluted M.

haemolytica genomic DNA with *rpoB* and *rpt2* primers. Lanes 1–7: gradient target DNA (10^{1} to 10^{-5} ng/μL); Lane 8: Negative control.

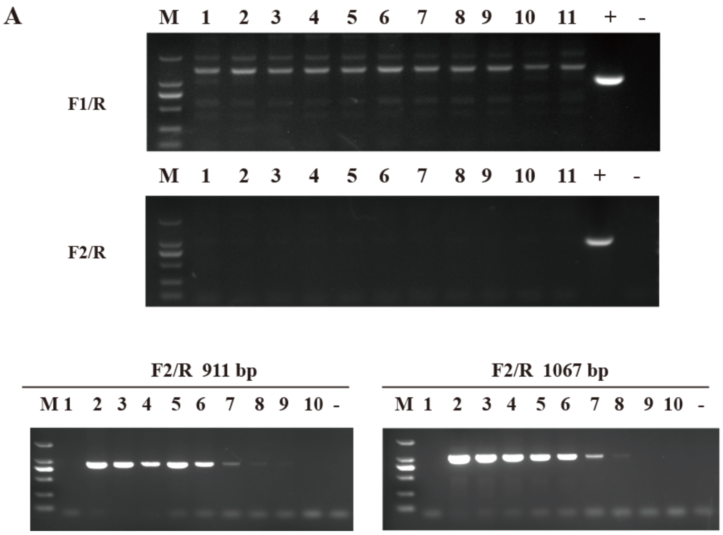

Figure S3. Universality and sensitivity of the rpt2-N primer. (A) Specificity comparison between F1/R and F2/R primer pairs. Lanes 1–11 represent genomic DNA from 11 ruminant bacterial strains stored in our laboratory (Table S1). (B) Sensitivity detection of the F2/R primer pair, showing amplicons of type II *rpt2* (left) and type I *rpt2* (right); 2: $1×10^{9}$ copies/25 μL；3：$1×10^{8}$ copies/25 μL；4：$1×10^{7}$ copies/25 μL；5：$1×10^{6}$ copies/25 μL；6：$1×10^{5}$ copies/25 μL；7：$1×10^{4}$ copies/25 μL；8：$1×10^{3}$ copies/25 μL；9：$1×10^{2}$ copies/25 μL；10：10 copy/25 μL.

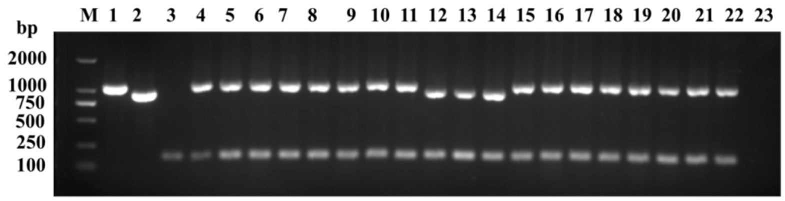

Figure S4. Gel electrophoresis results of the multiplex PCR system. Lanes 1 and 2: genome of Mh 39433 and genome of NCTC 10208 identified with *rpt2-N* primers. Lane 3: Mh 39433 detected *via Core* primers. Lane 4: Mh 39433 detected *via rpt2-N* primers and serotype primers. Lanes 5–22: Eighteen *M. haemolytica* isolates tested by multiplex PCR. Lane 23: Negative control.

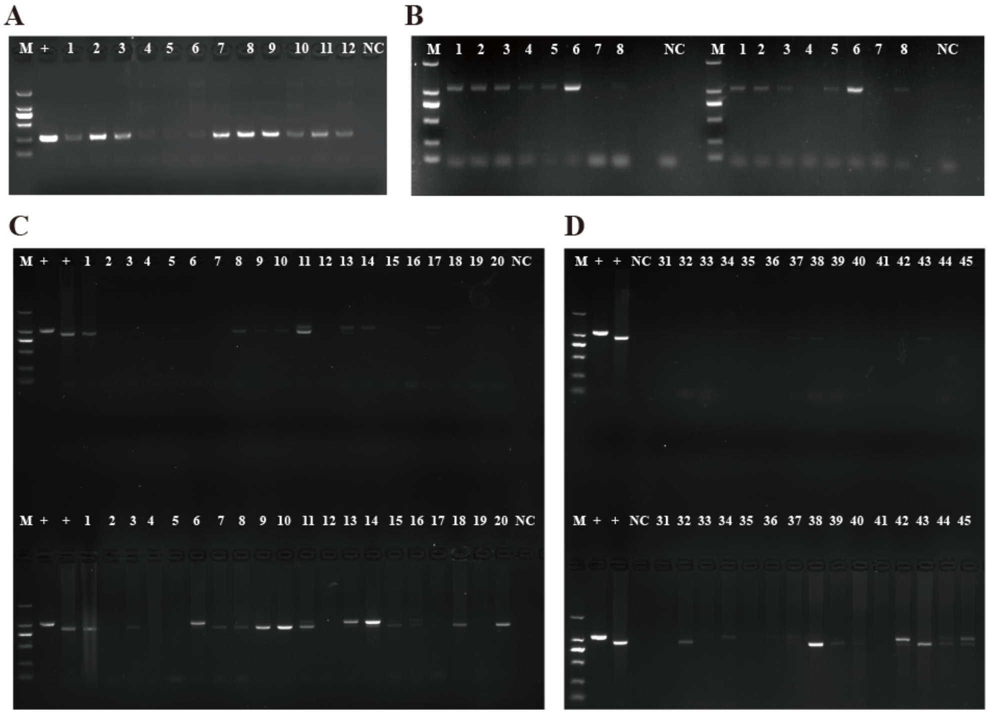

Figure S5. Epidemiological analysis of Mh in Chongqing sheep via *rpt2-N* primers. A: Four bacterial DNA extraction kits. Lanes 1–3: Solarbio YZ-D3350; 4–6: OMEGA D3350-00; 7–9: Tiangen DP302; 10–12: CWBIO CW0552S. B: Amplification comparison of two Taq enzymes. Two gels test eight identical clinical samples side by side. C–D: PCR detection results of *M. haemolytica* in nasal and pharyngeal swabs from two pens at a farm in Chongqing. Upper panel: pharyngeal swabs; lower panel: nasal swabs.
